# 1,1′-(Butane-1,4-di­yl)dipyridinium dibromide dihydrate

**DOI:** 10.1107/S1600536808000913

**Published:** 2008-01-18

**Authors:** Ming-Qiang Wu, Xin Xiao, Yun-Qian Zhang, Sai-Feng Xue, Qian-Jiang Zhu

**Affiliations:** a Key Laboratory of Macrocyclic and Supramolecular Chemistry of Guizhou Province, Guizhou University, Guiyang 550025, People’s Republic of China; b Institute of Applied Chemistry, Guizhou University, Guiyang 550025, People’s Republic of China

## Abstract

The organic cation in the title compound, C_14_H_18_N_2_^2+^·2Br^−^·2H_2_O, is situated on an inversion centre. The cations, anions and water mol­ecules are linked *via* O—H⋯Br, C—H⋯Br and C—H⋯O hydrogen bonds, and π–π stacking inter­actions between adjacent pyridine rings, with a centroid–centroid separation of 3.8518 (17) Å.

## Related literature

For general background, see: Day *et al.* (2000 ▶, 2002 ▶); Freeman *et al.* (1981 ▶); Kim *et al.* (2000 ▶).
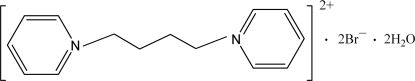


## Experimental

### 

#### Crystal data

C_14_H_18_N_2_^2+^·2Br^−^·2H_2_O
*M* *_r_* = 410.14
Monoclinic, 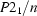

*a* = 11.0068 (13) Å
*b* = 7.1484 (8) Å
*c* = 12.0607 (13) Å
β = 111.602 (7)°
*V* = 882.30 (18) Å^3^
*Z* = 2
Mo *K*α radiation
μ = 4.60 mm^−1^
*T* = 293 (2) K
0.21 × 0.18 × 0.16 mm

#### Data collection

Bruker APEXII CCD area-detector diffractometer
Absorption correction: multi-scan (*SADABS*; Bruker, 2005 ▶) *T*_min_ = 0.393, *T*_max_ = 0.478
7189 measured reflections
1723 independent reflections
1483 reflections with *I* > 2σ(*I*)
*R*_int_ = 0.041

#### Refinement

*R*[*F* ^2^ > 2σ(*F* ^2^)] = 0.030
*wR*(*F* ^2^) = 0.074
*S* = 1.06
1723 reflections
92 parameters
3 restraints
H-atom parameters constrained
Δρ_max_ = 0.48 e Å^−3^
Δρ_min_ = −0.45 e Å^−3^

### 

Data collection: *APEX2* (Bruker, 2005 ▶); cell refinement: *SAINT* (Bruker, 2005 ▶); data reduction: *SAINT*; program(s) used to solve structure: *SHELXS97* (Sheldrick, 2008 ▶); program(s) used to refine structure: *SHELXL97* (Sheldrick, 2008 ▶); molecular graphics: *ORTEP-3 for Windows* (Farrugia, 1997 ▶); software used to prepare material for publication: *WinGX* (Farrugia, 1999 ▶).

## Supplementary Material

Crystal structure: contains datablocks global, I. DOI: 10.1107/S1600536808000913/fb2085sup1.cif

Structure factors: contains datablocks I. DOI: 10.1107/S1600536808000913/fb2085Isup2.hkl

Additional supplementary materials:  crystallographic information; 3D view; checkCIF report

## Figures and Tables

**Table 1:** Hydrogen-bond geometry (Å, °)

*D*—H⋯*A*	*D*—H	H⋯*A*	*D*⋯*A*	*D*—H⋯*A*
O1*W*—H1*WA*⋯Br1	0.85	2.48	3.323 (2)	172
O1*W*—H1*WB*⋯Br1^i^	0.85	2.53	3.375 (2)	175
C2—H2⋯Br1^ii^	0.93	2.86	3.664 (3)	145
C5—H5⋯O1*W*^iii^	0.93	2.55	3.376 (3)	148

Symmetry codes: (i) 
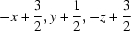
; (ii) 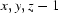; (iii) 
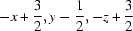
.

**Fig. 1.**
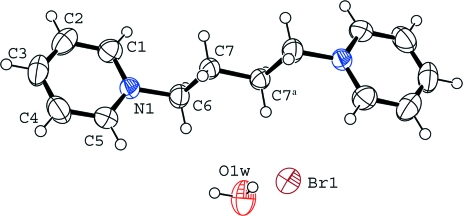
The molecular structure of (I) showing the atom-labelling scheme. Displacement ellipsoids are drawn at the 50% probability level. Symmetry code of C7a: -x + 1, -y, -z + 1.

## supplementary crystallographic information

### Comment

As a part of our ongoing investigation of polyaromatic compounds, we present a structure determination of the compound containing the pyridyl or alkyl groups that can be involved in intermolecular interactions with cucurbit[*n*]urils (CB[*n*]) (Freeman *et al.*, 1981; Day *et al.*, 2000; Day *et al.*, 2002; Kim *et al.*, 2000).

The organic cations in the title structure are situated on the inversion centres (Fig. 1) which coincide with the midpoint of the C7—C7^i^ bond [the symmetry code: (i) 1 - *x*,-*y*,1 - *z*]. The angle between the plane of the pyridine ring and the plane through C6,C7,C7^i^,C6^i^ chain is 86.57 (13)°. The anions and water molecules are linked *via* O—H···Br, C—H···Br, C—H···O hydrogen bonds (Table 1). In addition, the π···π stacking interactions occur between the adjacent pyridine rings, with the centroid-centroid separation being 3.8518 (17)Å [the symmetry code: (ii) 3/2 - *x*,-1/2 + *y*,1/2 - *z*].

### Experimental

A solution of 1,4-dibromine-butadinol (2.16 g, 0.01 mol) was added to a stirred solution of pyridine (1.98 g, 0.025 mol) in 1,4-dioxane (50 ml) at 110°C for 5 h. After cooling to room temperature, the mixture was filtered. The solid product was dissolved in 80 ml of water, and then set aside for three weeks to obtain colourless diamond-like crystals with average dimensions about 0.2 mm.

### Refinement

All the H atoms were located in the difference Fourier map. The H atoms attached to the carbon atoms were situated into the idealized positions and refined in a riding-atom approximation. The constraints: *C*—H_aryl_=0.93 and *C*—H_methylene_=0.97 Å; *U*_iso_(H) = 1.2*U*_eq_(C).

The positional parameters of water H atoms were restrained with the distances O—H equal to 0.85 (1)Å while with the distance between both H atoms equal to 1.35 (2) Å. The water H atoms were refined as riding with *U*_iso_(H) = 1.2*U*_eq_(O).

### Crystal data

**Table:**

C_14_H_18_N_2_^2+^·2Br^–^·2H_2_O	*F*_000_ = 412
*M**_r_* = 410.14	*D*_x_ = 1.544 Mg m^−^^3^
Monoclinic, *P*2_1_/*n*	Mo *K*α radiation λ = 0.71073 Å
Hall symbol: -P 2yn	Cell parameters from 1730 reflections
*a* = 11.0068 (13) Å	θ = 0.5–0.6º
*b* = 7.1484 (8) Å	µ = 4.60 mm^−^^1^
*c* = 12.0607 (13) Å	*T* = 293 (2) K
β = 111.602 (7)º	Diamond, colourless
*V* = 882.30 (18) Å^3^	0.21 × 0.18 × 0.16 mm
*Z* = 2	

### Data collection

**Table:**

Bruker CCD area-detector diffractometer	1723 independent reflections
Radiation source: fine-focus sealed tube	1483 reflections with *I* > 2σ(*I*)
Monochromator: graphite	*R*_int_ = 0.041
*T* = 293(2) K	θ_max_ = 26.0º
φ and ω scans	θ_min_ = 2.1º
Absorption correction: multi-scan(SADABS; Bruker, 2005)	*h* = −13→13
*T*_min_ = 0.393, *T*_max_ = 0.478	*k* = −8→8
7189 measured reflections	*l* = −14→14

### Refinement

**Table:**

Refinement on *F*^2^	Secondary atom site location: difference Fourier map
Least-squares matrix: full	Hydrogen site location: difference Fourier map
*R*[*F*^2^ > 2σ(*F*^2^)] = 0.030	H-atom parameters constrained
*wR*(*F*^2^) = 0.074	*w* = 1/[σ^2^(*F*_o_^2^) + (0.0377*P*)^2^ + 0.1891*P*] where *P* = (*F*_o_^2^ + 2*F*_c_^2^)/3
*S* = 1.06	(Δ/σ)_max_ = 0.001
1723 reflections	Δρ_max_ = 0.48 e Å^−^^3^
92 parameters	Δρ_min_ = −0.45 e Å^−^^3^
3 restraints	Extinction correction: SHELXL97 (Sheldrick, 2008), Fc^*^=kFc[1+0.001xFc^2^λ^3^/sin(2θ)]^-1/4^
38 constraints	Extinction coefficient: 0.073 (3)
Primary atom site location: structure-invariant direct methods	

### Special details

**Table:**

Geometry. All e.s.d.'s (except the e.s.d. in the dihedral angle between two l.s. planes) are estimated using the full covariance matrix. The cell e.s.d.'s are taken into account individually in the estimation of e.s.d.'s in distances, angles and torsion angles; correlations between e.s.d.'s in cell parameters are only used when they are defined by crystal symmetry. An approximate (isotropic) treatment of cell e.s.d.'s is used for estimating e.s.d.'s involving l.s. planes.
Refinement. Refinement of *F*^2^ against ALL reflections. The weighted *R*-factor *wR* and goodness of fit *S* are based on *F*^2^, conventional *R*-factors *R* are based on *F*, with *F* set to zero for negative *F*^2^. The threshold expression of *F*^2^ > σ(*F*^2^) is used only for calculating *R*-factors(gt) *etc*. and is not relevant to the choice of reflections for refinement. *R*-factors based on *F*^2^ are statistically about twice as large as those based on *F*, and *R*- factors based on ALL data will be even larger.

### Fractional atomic coordinates and isotropic or equivalent isotropic displacement parameters (Å^2^)

**Table:**

	*x*	*y*	*z*	*U*_iso_*/*U*_eq_	
C1	0.6134 (3)	0.1783 (4)	0.2544 (2)	0.0512 (6)	
H1	0.5228	0.1803	0.2175	0.061*	
C2	0.6896 (3)	0.1660 (4)	0.1867 (2)	0.0602 (7)	
H2	0.6509	0.1598	0.1040	0.072*	
C3	0.8220 (3)	0.1629 (4)	0.2408 (3)	0.0627 (8)	
H3	0.8741	0.1553	0.1953	0.075*	
C4	0.8782 (3)	0.1710 (5)	0.3631 (3)	0.0655 (8)	
H4	0.9686	0.1687	0.4012	0.079*	
C5	0.7990 (3)	0.1826 (4)	0.4285 (2)	0.0515 (6)	
H5	0.8359	0.1870	0.5114	0.062*	
C6	0.5845 (2)	0.1926 (3)	0.4453 (2)	0.0458 (6)	
H6A	0.5090	0.2711	0.4059	0.055*	
H6B	0.6329	0.2462	0.5229	0.055*	
C7	0.5400 (2)	−0.0023 (3)	0.46040 (19)	0.0389 (5)	
H7A	0.6155	−0.0826	0.4959	0.047*	
H7B	0.4875	−0.0534	0.3830	0.047*	
N1	0.66903 (18)	0.1874 (3)	0.37309 (16)	0.0393 (5)	
O1W	0.6333 (2)	0.5305 (3)	0.7900 (2)	0.0762 (6)	
H1WA	0.6461	0.4149	0.8070	0.091*	
H1WB	0.6735	0.5522	0.7437	0.091*	
Br1	0.69404 (3)	0.09270 (4)	0.88751 (2)	0.05201 (16)	

### Atomic displacement parameters (Å^2^)

**Table:**

	*U*^11^	*U*^22^	*U*^33^	*U*^12^	*U*^13^	*U*^23^
C1	0.0517 (15)	0.0538 (15)	0.0443 (13)	0.0009 (12)	0.0133 (12)	0.0042 (12)
C2	0.087 (2)	0.0545 (16)	0.0457 (14)	0.0055 (15)	0.0326 (15)	0.0043 (13)
C3	0.086 (2)	0.0498 (15)	0.077 (2)	−0.0030 (15)	0.0598 (19)	0.0006 (15)
C4	0.0500 (17)	0.0680 (18)	0.087 (2)	−0.0151 (15)	0.0353 (16)	−0.0133 (17)
C5	0.0428 (15)	0.0613 (16)	0.0487 (14)	−0.0095 (12)	0.0148 (12)	−0.0098 (13)
C6	0.0466 (14)	0.0484 (14)	0.0492 (13)	−0.0010 (11)	0.0253 (12)	−0.0008 (11)
C7	0.0359 (12)	0.0449 (13)	0.0359 (11)	−0.0006 (10)	0.0133 (10)	0.0009 (10)
N1	0.0417 (12)	0.0374 (11)	0.0421 (10)	−0.0042 (8)	0.0193 (9)	−0.0015 (8)
O1W	0.0797 (15)	0.0731 (13)	0.0922 (15)	0.0221 (12)	0.0509 (14)	0.0232 (13)
Br1	0.0555 (2)	0.0537 (2)	0.0485 (2)	0.00237 (12)	0.02118 (15)	0.00144 (11)

### Geometric parameters (Å, °)

**Table:**

C1—N1	1.335 (3)	C5—H5	0.9300
C1—C2	1.372 (4)	C6—N1	1.490 (3)
C1—H1	0.9300	C6—C7	1.510 (3)
C2—C3	1.359 (4)	C6—H6A	0.9700
C2—H2	0.9300	C6—H6B	0.9700
C3—C4	1.375 (4)	C7—C7^i^	1.519 (4)
C3—H3	0.9300	C7—H7A	0.9700
C4—C5	1.377 (4)	C7—H7B	0.9700
C4—H4	0.9300	O1W—H1WA	0.8507
C5—N1	1.338 (3)	O1W—H1WB	0.8454
			
N1—C1—C2	120.1 (3)	N1—C6—H6A	109.6
N1—C1—H1	120.0	C7—C6—H6A	109.6
C2—C1—H1	120.0	N1—C6—H6B	109.6
C3—C2—C1	119.9 (3)	C7—C6—H6B	109.6
C3—C2—H2	120.1	H6A—C6—H6B	108.1
C1—C2—H2	120.1	C6—C7—C7^i^	110.2 (2)
C2—C3—C4	119.6 (2)	C6—C7—H7A	109.6
C2—C3—H3	120.2	C7^i^—C7—H7A	109.6
C4—C3—H3	120.2	C6—C7—H7B	109.6
C3—C4—C5	119.2 (3)	C7^i^—C7—H7B	109.6
C3—C4—H4	120.4	H7A—C7—H7B	108.1
C5—C4—H4	120.4	C1—N1—C5	121.2 (2)
N1—C5—C4	120.1 (3)	C1—N1—C6	119.3 (2)
N1—C5—H5	120.0	C5—N1—C6	119.4 (2)
C4—C5—H5	120.0	H1WA—O1W—H1WB	104.9
N1—C6—C7	110.37 (19)		
			
N1—C1—C2—C3	0.1 (4)	C2—C1—N1—C6	−177.4 (2)
C1—C2—C3—C4	0.4 (5)	C4—C5—N1—C1	1.0 (4)
C2—C3—C4—C5	−0.1 (5)	C4—C5—N1—C6	177.7 (3)
C3—C4—C5—N1	−0.6 (4)	C7—C6—N1—C1	81.1 (3)
N1—C6—C7—C7^i^	176.9 (2)	C7—C6—N1—C5	−95.6 (3)
C2—C1—N1—C5	−0.8 (4)		

Symmetry codes: (i) −*x*+1, −*y*, −*z*+1.

### Hydrogen-bond geometry (Å, °)

**Table:**

*D*—H···*A*	*D*—H	H···*A*	*D*···*A*	*D*—H···*A*
O1W—H1WA···Br1	0.85	2.48	3.323 (2)	172
O1W—H1WB···Br1^ii^	0.85	2.53	3.375 (2)	175
C2—H2···Br1^iii^	0.93	2.86	3.664 (3)	145
C5—H5···O1W^iv^	0.93	2.55	3.376 (3)	148

Symmetry codes: (ii) −*x*+3/2, *y*+1/2, −*z*+3/2; (iii) *x*, *y*, *z*−1; (iv) −*x*+3/2, *y*−1/2, −*z*+3/2.
